# In vitro and in vivo MRI imaging and photothermal therapeutic properties of Hematite (α-Fe_2_O_3_) Nanorods

**DOI:** 10.1007/s10856-021-06636-1

**Published:** 2022-01-12

**Authors:** Aanisa Gulzar, Nowsheena Ayoub, Jaffar Farooq Mir, Amer M. Alanazi, M. A. Shah, Arif Gulzar

**Affiliations:** 1 Laboratory for Multifunctional Nanomaterials, P.G Department of Physics, National Institute of Technology Srinagar, Hazratbal, Srinagar, J&K 190006 India; 2 Pharmaceutical Chemistry Department, College of Pharmacy, King Saud University, Riyadh, 11451 Saudi Arabia; 3 Med X Institute, School of Biomedical Engineering Shanghai Jiao Tong University, Shanghai, 200030 China; 4 Hevesy Laboratory, Center for Nuclear Technologies, DTU Health Tech, 4000 Roskilde, Denmark

**Keywords:** Nanorods, PEGylation, PTT, MRI, Imaging

## Abstract

Herein we report synthesis of hematite (α-Fe_2_O_3_) nanorods by calcinating hydrothermally synthesized goethite nanorods at 5000C. The structural, optical and MRI imaging guided cancer therapeutic properties of fabricated nanorods have been discussed in this manscript. FESEM and TEM imaging techniques were used to confirm the nanorod like morphology of as prepared materials. As we know that Fe_2_O_3_ nanorods with size in the range of 25–30 nm exhibit super magnetism. After coating with the PEG, the as prepared nanorods can be used as *T*_*2*_ MR imaging contrast agents. An excellent *T*_*2*_ MRI contrast of 38.763 mM^–1^s^–1^ achieved which is highest reported so far for α-Fe_2_O_3_. Besides the as prepared nanorods display an excellent photothermal conversion efficiency of 39.5% thus acts as an excellent photothermal therapeutic agent. Thus, we envision the idea of testing our nanorods for photothermal therapy and MR imaging application both in vitro and in vivo, achieving an excellent *T*_*2*_ MRI contrast and photothermal therapy effect with as prepared PEGylated nanorods.

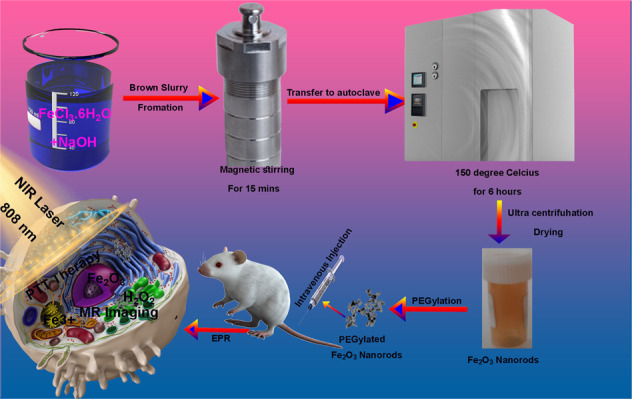

## Introduction

In recent times, the fabrication of nanostructures with different morphologies has received considerable attention due to their unique properties and diverse applications [1, 2]. The physical, chemical, and optical properties of nanomaterials have significant dependence upon the morphology of materials [3–5]. The synthesis of nanostructures other than spherical morphologies has attracted much attention from scientists for their morphology dependent applications in biomedical, sensing and catalysis [5–7]. Compared to nanospheres, non-spherical morphologies of iron oxide nanostructures have shown great advantages in many applications and have been extensively studied and explored in various application fields because of their low cost, nontoxicity, and biocompatibility [8–10].

**Scheme 1:**
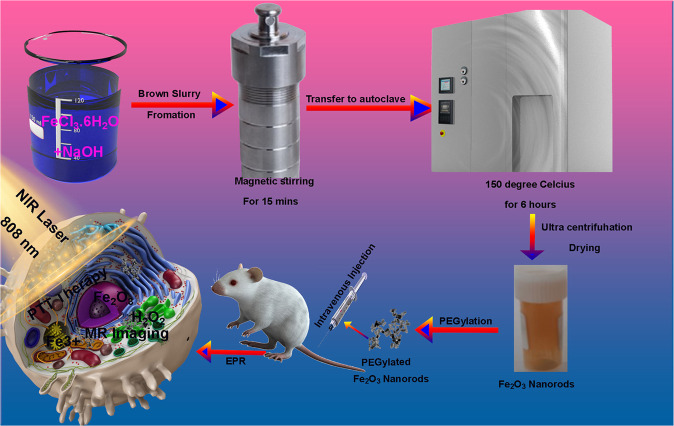
Schematic illustration of synthesis, PPT and MRI imaging Property of as prepared Fe_2_O_3_ nanorods

Iron belongs to the transition elements group which has 26 protons in nucleus. The most stable isotopes of iron are ^54^Fe, ^56^Fe, ^57^Fe and ^58^Fe. Since iron has eight electrons in the valence shell and due to the electronegative nature of oxygen, it exhibits bi-valency and tri-valency to form bivalent and trivalent compounds [11]. Among the various oxides of iron, Fe_2_O_3_ (Hematite) is the most common and stable oxide having a great magnetic character. The crystal structure of hematite is quite alike to α-Al_2_O_3_-corundum. In its hexagonal close packed structure, the hexagonal site is occupied by oxygen ions and octahedral sites by iron ions. Since it is not a typical ferromagnetic, so its antiferromagnetic nature is of huge interest [12, 13]. To date, numerous methods have been used to produce iron oxide nanostructures [14]. Nanostructures with different morphologies of these oxides have already been reported [15, 16]. Among these morphologies, nanorod-like shape morphology has been the specific focus of interest and attention because of its regular geometry [17, 18]. Nanomaterials with nanorod like morphology have shown superior performance in various application fields such as MRI imaging etc. [19, 20]. Unfortunately, most of the synthesis methods use costly precursors, templates, surfactants, and high temperature. Therefore, there is need to fabricate nanostructures via a simple and economical methods and that too at low temperatures. Hydrothermal route when used at low temperatures satisfies above criteria. Herein we synthesized the α-Fe_2_O_3_ via a simple facile hydrothermal method as shown in Scheme 1.

For the in vitro and in vivo application of α-Fe_2_O_3_, the PEG coating is critical step to improve colloidal stability and biocompatibility. So, we coated our nanorods with the PEG to make them biocompatible. Photothermal therapy is a kind of therapy based on increasing the temperature of tumorous cells above 42 °C. To this aim, cells must be illuminated with a laser, and the energy of the radiation is transformed in heat. Usually, the employed radiation belongs to the near-infrared radiation range. At this range, the absorption and scattering of the radiation by the body is minimal. To improve the efficacy and selectivity of the energy-to-heat transduction, a light-absorbing material, the photothermal agent, must be introduced into the tumor. Herein we tested our α-Fe_2_O_3_ nanorods for PTT efficiency at a laser power density of 0.5 Wcm^−2^ for 5 min with irradiation by an 808 nm Laser. Besides, as we know magnetic resonance imaging (MRI) has become an integral part of modern clinical imaging due to its non-invasiveness and versatility in providing tissue and organ images with high spatial resolution [21–27]. With the current MRI advancement, MRI imaging probes with suitable biocompatibility, good colloidal stability, enhanced relaxometric properties and advanced functionalities are highly demanded. As such, MRI contrast agents (CAs) have been an extensive research and development area [28–30]. In the recent years, different inorganic-based nanoprobes comprising inorganic magnetic nanoparticles (MNPs) with an organic functional coating have been engineered to obtain a suitable contrast enhancement effect [31–34]. In this manuscript, we report synthesis of hematite nanorods by thermal conversion of hydrothermally synthesized goethite nanorods. The structural and optical properties of fabricated nanorods have been discussed in this manuscript, with special focus on the application of the PEG conjugated α-Fe_2_O_3_ nanorods for in vitro and in vivo photothermal therapy and MRI imaging. We report excellent in vitro and in vivo photothermal therapeutic effect by α-Fe_2_O_3-_PEG nanorods with an excellent thermal conversion efficiency of 39.5% not reported so far. Besides that, we also report an excellent MRI imaging contrast of 38.763 mM^–1^ s^–1^ which is the highest reported MR contrast for any pure iron oxide to the best of our knowledge.

## Experimental section

### Materials

FeCl_3_.6H_2_O, NaOH, Ethanol, Acetone, mPEG, deionized water (DI).PBS, DMEM, DAPI. All precursors were used without further filtration or purification. Propidium iodide (PI),3-(4,5-dimethyl-2 thiazolyl)-2,5-diphenyl-2H-tetrazolium bromide (MTT), Calcein AM, and 2,7-dichlorofluorescein diacetate (DCFH-DA) were procured from Sigma-Aldrich Co. LLC.

### Hydrothermal fabrication of nanorods

In procedure, 0.25 mol L^−1^ of FeCl_3_ yellow color solution is mixed with 2 mol L^−1^ of NaOH solution which leads to formation of a brown color slurry like substance to which 8.0 mL of deionized water is added drop by drop separately under the magnetic stirring at room temperature for 15 min. The brown colored paste obtained is transferred into a Teflon lined autoclave with capacity of 60 mL out of which 40 mL was filled. The autoclave was firstly dried with airstream after being washed with Acetone Ethanol and DI water for 15 min each before solution was transferred into it. The autoclave was then placed in a muffle furnace whose temperature was allowed to raise up to 150 °C with heating rate of 2 degrees per minute and kept a at constant temperature for 6 h and was then allowed to cool down naturally to room temperature [35]. The product obtained was then cleaned with DI water followed by centrifugation for 30 min at 10000 rpm and then dried at 80 ^o^C for 24 hours. After 24 hours the sample was annealed at 500 °C.

### Modification of nanorods with PEG

As prepared nanorods with a concentration of 0.5 mg/mL^−1^ were dissolved in deionized water at room temperature. To this solution an 0.5 mg/mL^−1^ solution of PEG was added dropwise and left to stir at room temperature for 2 h, followed by ultracentrifugation to get the final product.

### Characterization

The X- ray diffractometer (XRD, RIGAKU) and Raman spectrometer (REINSHAW) were used to identify the crystal structure and phase purity. Field emission scanning electron microscopy (FESEM, ZEISS GEMINI 500) was used to confirm the shape morphology of sample. FESEM examination was done after annealing the sample at 500 °C of sample. UV-VIS-NIR spectrometer (SHIMADZU UV-3600) was employed to identify optical properties and respective band gap.

### In vitro Photothermal Studies

In brief, in a 96-well plate over a concentration range (400, 200, 100, 25, and 0 ppm), 200 μL of an aqueous solution of α-Fe_2_O_3_-PEG was added to each corresponding well of cells. At that point, an 808 nm NIR laser (0.5 W cm^−2^) was established, and cells were irradiated with the NIR laser for 5 min, followed by cooling to room temperature. The infrared image of the samples and temperature were tracked through an infrared camera (R300SR-HD, NEC). Using Equation S1, S2 and S3, the photothermal conversion efficiency (η) was calculated.

### Cell viability and cellular uptake experiments

The cell viability of PEGylated Fe_2_O_3_ was confirmed by MTT methods, in which the L929 fibroblast cells were used as specialized cells for detection of cell viability. Briefly, the L929 cells were incubated in 96-well plate (6000–7000/well) at 37 °C in 5% CO_2_ for 24 h. Then, solutions of PEGylated Fe_2_O_3_ at several concentrations (15.6, 31.2, 62.5, 125, 250, and 500 µg/mL) were added into the cells and the treated cells were kept on cultivating for 24 h. Afterwards, the 15 μL of MTT solution (5 mg/mL) was added into each well and the treated cells were kept on incubating at 37 °C for another 3 h. Finally, 120 μL of DMSO was supplied into each well and the data of cell viability was measured by Microplate reader at 490 nm.

The cellular uptake process of PEGylated Fe_2_O_3_ nanoparticles was as follows: To begin with, the HeLa cells were cultivated in the 6-well plate to form a monolayer cell. Then PEGylated Fe_2_O_3_ solution (500 µg/mL) was added into the plate and the cells of plate continued incubating in different time (0.5, 1, and 3 h). Next, the cells were washed with PBS several times and stained with DAPI (25 µg/mL) for 5 min so that the dye could mark the nucleus of cells. At last, the cells were fixed by 2.5% glutaraldehyde (1 mL) for 10 min and observed by using CLSM.

### In vitro Cytotoxicity and photothermal therapy

The in vitro cytotoxicity of the samples may well be established through MTT methods. At first, we cultivated the HeLa cells in a 96-well plate with a density of 7000 per well, which was placed into an incubator (37 1 C, 5% CO_2_) for a period of 24 h. Next, PEGylated Fe_2_O_3_, with various concentrations of 0, 15.6, 31.2, 62.5, 125, 250, and 500 mg mL^−1^ were injected into the 96-well plate. Then, the cells of the 96-well plate were treated as follows: Control, NIR, PEGylated Fe_2_O_3_, PEGylated Fe_2_O_3_ + NIR (NIR laser: 808 nm, 0.5 W cm^−2^). Before NIR laser irradiation, each sample was already supplied and rested to incubate for about 4 h for completing the cellular uptake. Afterwards, the treatment of cells was done in the same way as in the MTT methods as per the cell viability assay on L929 cells.

### In vitro and in vivo MR imaging

Two BALB/c nude and 40 C57 mice, 4–6 weeks of age and weighting 18–22 g, were purchased from shanghai LAC laboratory animal Co., Ltd., and housed in an SPF grade animal centre. The use of all mice/mouse in this study complied with the current ethical considerations: Approval of institutional Animal Care and Use Committee of Shanghai Jiao Tong University. The mice were anesthetized by isoflurane and 2 × 10^6^ Lewis cells suspended in 100 μL saline were transplanted into the mice subcutaneous. A 0.5 T MR imaging magnet was employed for testing the in vitro and in vivo MR imaging. The PEGylated Fe_2_O_3_ were dispersed in water carrying different concentrations of Mn and Gd. The *T*_*2*_ measurements were done before and after injection of PEGylated Fe_2_O_3_ (100 mL, 4 × 10^–3^ M). Lastly, the *r*_*2*_ relativity values were determined by the curve fitting of *1/T*_*2*_ relaxation time (s^–1^) versus the total concentration of Fe (mM).

### In vivo animal studies

Female blab mic 4–6 weeks of age and weighting 18–22 g, were purchased from shanghai LAC laboratory animal Co Ltd., and housed in an SPF grade animal centre. All the animal experiments were conducted in compliance with the specifications of the use of all mice/mouse in this study complied with the current ethical considerations: Approval of institutional Animal Care and Use Committee of Shanghai Jiao Tong University. We rooted the U14 cells in the left axilla in each mouse; after this, mice in group A were indiscriminately separated into groups of five: control group (group I), NIR irradiated group (group II), PEGylated Fe_2_O_3_ (group III), PEGylated Fe_2_O_3_ with NIR irradiation group (group IV). 0.5 W cm^−2^ was the power density of the 808 nm laser that was used. Before intravenous injection of the mice, each sample was dissolved in normal saline. Through intravenous injection, these samples were inoculated in each mouse (500 mg µL^−1^, 100 µL) apart from that of group I and II mice, which were administered with 100 µL of regular saline as soon as the tumor location of the mice increased up to 5 mm^3^. After 2 h of the dose, the tumor spots of groups II, IV, and V were subjected to 808 nm laser for a period of 10 min. Subsequently, the tumor sizes of group’s I–V were gauged through a caliper every 2nd day; in addition, the tumor volume of each group was calculated as per the following formula:

1 $${{{{{\mathrm{V}}}}}} = {{{{{\mathrm{L}}}}}} \times {{{{{\mathrm{W}}}}}}^2/2$$

where the volume, length, and width of the tumor are denoted by V, L, and W, respectively. In addition, V/V_0_ gave us the relative tumor volume (V_0_ denotes the original tumor volume of the mice).

## Results and discussions

### Morphology

**Fig. 1:**
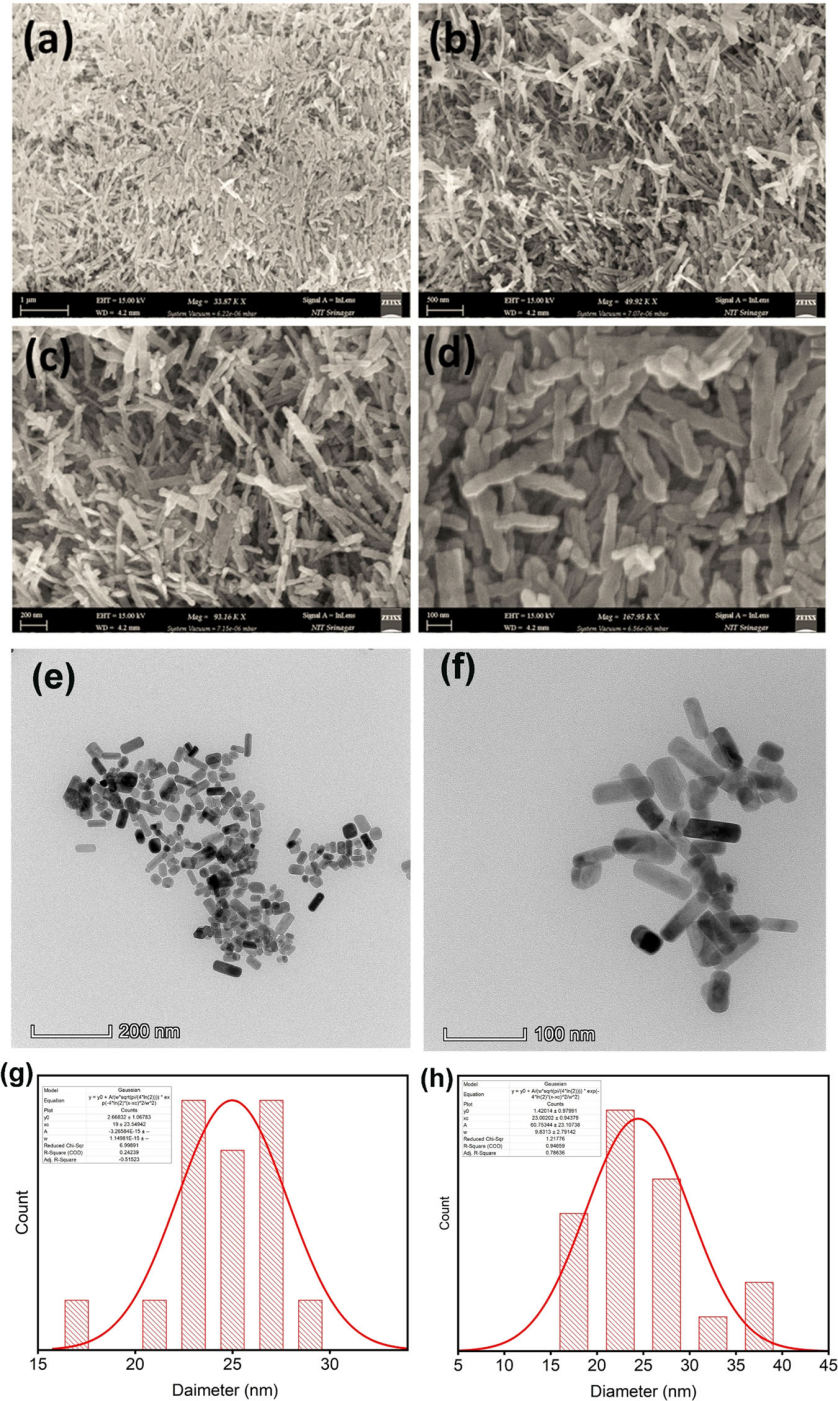
**a**–**d** FESEM images of hematite nanorods fabricated via hydrothermal synthesis, (**e**, **f**) TEM images of hematite nanorods, (**g**, **h**) hydrodynamic size distribution of α-Fe_2_O_3_ nanorods

**Fig. 2:**
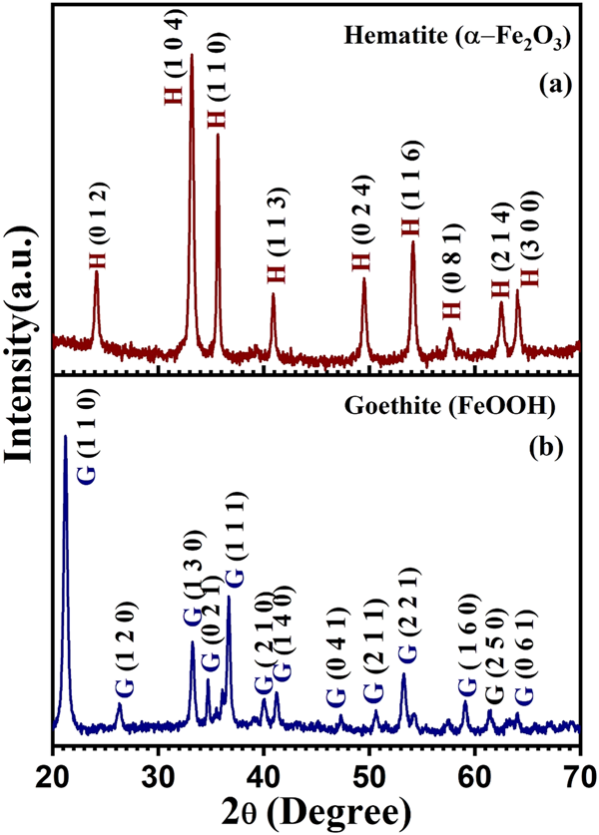
X-ray diffraction patterns: (**a**) Hematite nanorods annealed at 500 ^o^C (**b**) Goethite (unannealed) sample

The morphology of as-synthesized annealed sample was determined by FESEM and TEM imaging. Figure 1a–d shows FESEM images which depicts nanorods like morphology of the sample. The high-magnification image of iron oxide nanorods is shown in Fig. 1d which reveals nanorods fabricated are dense. Further by using the TEM imaging Fig. 1e–g we can see the morphology of the as prepared sample as nanorods with an average diameter of 19 nm as determined by the hydrodynamic size distribution and Fig. 1f–h, shows the monodisperse PEGylated nanorods with an average diameter of 23 nm as determined by the hydrodynamic size distribution. The increase in the diameter of few nanometers can be attributed to the annealing process. To confirm the phase purity of as prepared nanorods the XRD analysis was carried out. XRD patterns are shown in Fig. 2. A substantial difference in phase was found in the XRD patterns of the un-annealed and annealed samples. Most of the diffraction peaks of un-annealed sample can be assigned to Goethite (FeOOH) [36]. The diffraction positioning’s of the Goethite peaks are observed at 21.23°, 26.29°, 33.29°, 34.73°, 36.69°, 39.99°, 41.23°,47.20°,50.71°,53.27°,59.04°, 61.4° and 63.99° which have been indexed to their respective planes as shown in Fig. 2. The intensity of the (111) and (130) is relatively high compared other peaks. The diffraction peaks located at 24.12°, 33.08°, 35.66°,40.91°,49.46°,54.21°, 57.60°, 62.46° and 63.99° have been assigned to the hematite (α- Fe_2_O_3_) [37]. The intensity of the (104) and (110) is relatively high compared other peaks of hematite. Goethite phase transforms into hematite when calcinated at ∼500 °C. Furthermore, the calcinated sample revealed higher intensities than the un-annealed sample.

Further the crystalline size and strain of the as prepared nanorod was calculated by using Debye–Scherrer method.

The method was employed to estimate the crystallite size by utilizing the XRD data. The expansion of Bragg peaks helps in predicting crystallite size using Debye-Scherrer equation [38].

2 $$D = \frac{{K\lambda }}{{\beta {{{{{\mathrm{cos}}}}}}\theta }}$$

where D in Eq. 2. Represents crystallite size, K is called shape factor (~0.9), λ represents the source-radiation wavelength (0.154 nm), β represents full width at half maximum (FWHM) of the corresponding Bragg peaks. The crystallite size of un-annealed sample taken as mean was calculated using above equation and came out to be 19 nm and of annealed samples was found to be 23 nm which corresponds to the TEM results. This increase in crystallite can be attributed to the calcination process.

Crystallite size was also calculated from Williamson Hall deformation model (UDM) by using below equation:

3 $$\beta {{{{{\mathrm{cos}}}}}}\theta = \frac{{K\lambda }}{D} + 4\varepsilon {{{{{\mathrm{sin}}}}}}\theta$$

where ε represents the amount of tensile (positive slope) or compressive strain (negative slope).

Furthermore, Williamson Hall analysis is significant in determining the nature of strain [28]. UDM plot for un-annealed and annealed sample is shown in Fig. S1. The intercept on Y-axis from the linearly fitted line of (βcosθ vs 4sinθ) was used to determine the crystallite size. The negative slope in case of un-annealed sample indicates the presence of compressive strain and the annealed sample revealed a positive slope which indicates tensile strain present in the sample. The compressive strain in annealed sample can be attributed to annealing of as fabricated sample at 500 ^0^C. The calculated strains of un-annealed and annealed samples was found to be −3.1 × 10^−3^ and 1.6 × 10^−4^ [39, 40].

**Fig. 3:**
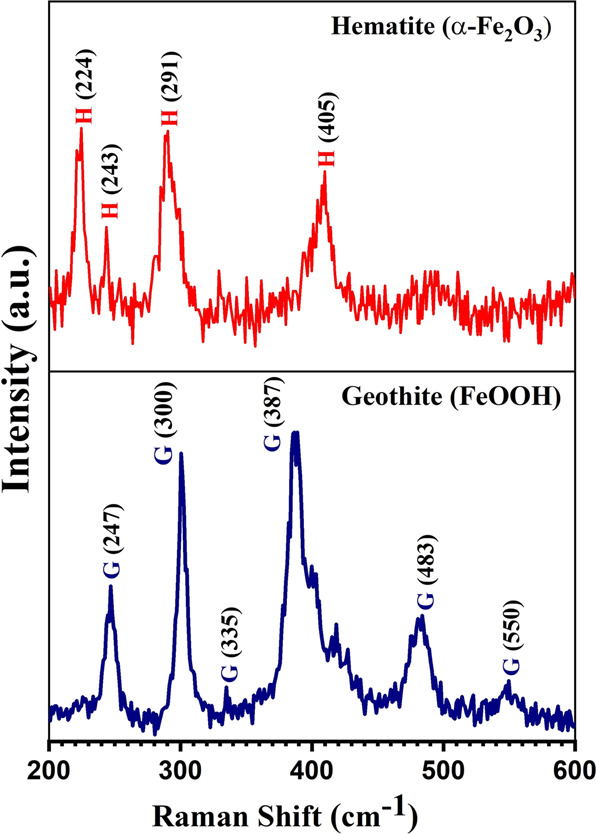
Raman spectra of nanorods: Goethite phase (un-annealed sample Hematite phase (annealed sample)

**Fig. 4:**
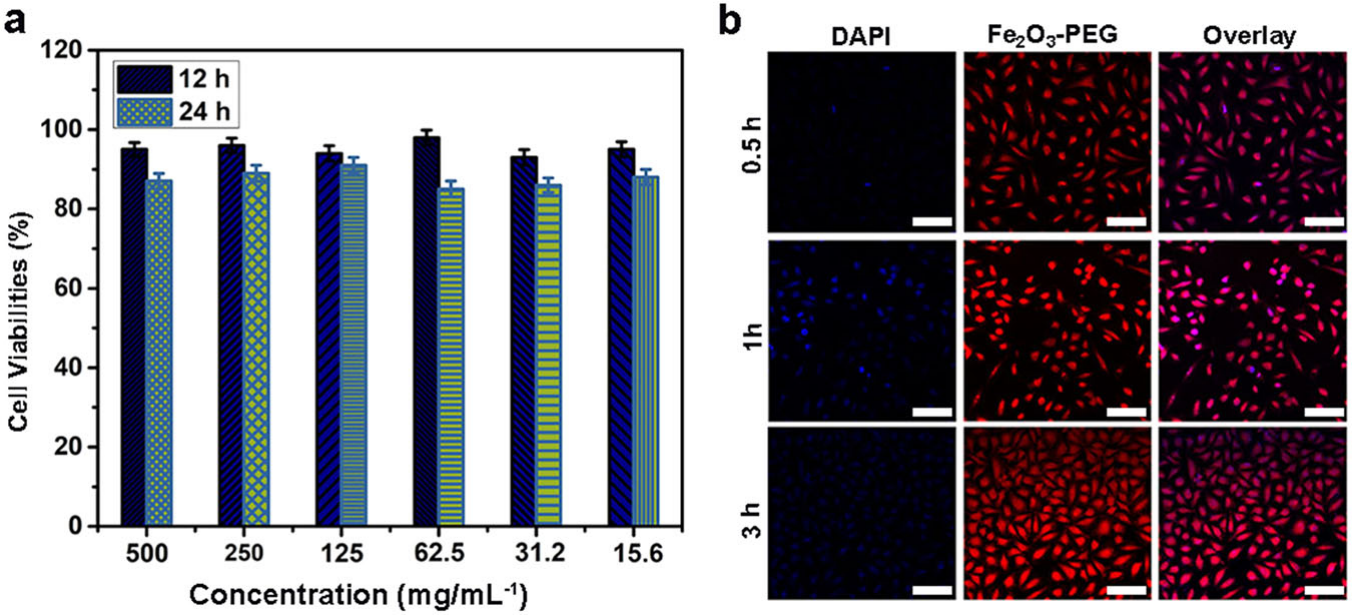
**a** Cell viability of PEGylated α- Fe_2_O_3_ (**b**) Cellular uptake of PEGylated α- Fe_2_O_3_ in Hela cells. Scale bar 75 µm. Statistical analysis was performed using Student’s two-tailed *t* test (*P<0.05)

Raman spectroscopy was employed to further confirm the phase purity of the as prepared nanorods. The un-annealed sample Fig. 3 shows the different peaks approximately located at 247 cm^−1^, 300 cm^1^, 335 cm^−1^, 387 cm^−1^, 483 cm^−1^ and 550 cm^−1^ respectively. All the peaks were found to be the characteristic peaks of Goethite phase [41]. The annealed sample Fig. 4 showed the various peaks which are roughly located at 224 cm^−1^, 243 cm^−1^, 291 cm^−1^ and 405 cm^−1^ respectively. The peaks were found to be the characteristic peaks of Hematite [37]. The FT-IR analysis of sample was carried Fig. S2 to confirm the successful conjugated of the PEG on the nanorods. The IR band at 1730 cm^−1^ is credited to the −C=O of PEG and the band due to C–O stretching mode were merged in a very broad envelop centered on 1268 and 1007 cm^−1^ thus confirming the successful conjugated of PEG on the α- Fe_2_O_3_ nanorods.

### Optical properties

Before in vitro and in vivo analysis, the optical properties and the solubility of the nanorods in the aqueous media should be evaluated. The optical properties of hydrothermally synthesized hematite nanorods annealed at 500 °C were determined by employing UV-VIS spectroscopy shown in Fig S3. The absorption edge of annealed sample is about ~740 nm as can be seen in Fig S3a. It further reveals a high visible light adsorption as the absorbance band of sample can be seen in visible region [42–44]. Fig. S3b shows the reflectance spectra. Tauc’s equation (Eq. 4) was used to calculate Band gap energy of the sample:

4 $$\alpha h\nu = {{{{{\mathrm{A}}}}}}\left( {h\nu --{{{{{\mathrm{E}}}}}}_{{{{{\mathrm{g}}}}}}} \right)^{{{{{\mathrm{n}}}}}}$$

where A is a constant, α represents absorption coefficient, ν represents light frequency, and Eg represents the band gap.

The optical band gap was found via tauc plot Fig. S4. The band gap energy as calculated was found to be ~2.08 eV which is consistent with the literature and falls in the band gap range of typical Hematite (1.9eV–2.3 eV) [40]. Furthermore, the optical properties of hematite nanorods also reveal that this type of morphology can significantly be used in NIR and visible light driven applications. The nanorods were found to be highly soluble when coated with PEG in different media as shown in Fig. S5a. Here we can observe that PEGylated nanorods had no sedimentation over a time period of 0–72 h. While as uncoated nanorods showed strong sedimentation after 30 min. Thus, confirming our PEGylated nanorods were high soluble in different cellular media. Zeta potential values where recorded for the final nanocomposite in various biological mediums to further confirm the solubility and a zeta potential values of −37 mV, −32 mV and −31.2 mV in H2O, PBS and FBS confirm the high solubility of nanocomposite as show in Fig. S5b.

### In vitro cell viability and cytotoxicity

Before in vitro and in vivo Photothermal therapy and MR imaging we evaluated the cellular viability and cellular uptake of nanorods in L929 and Hela cells. As an important data, the biocompatibility of the as-obtained PEGylated α-Fe_2_O_3_ nanorods s must be evaluated before actual application in biological studies. The cell viabilities of the L929 cells after incubation with different concentration for 12–24 h are shown in Fig. 4a. The nanocomposite displays high cell viability of 85–98% in an overall concentration range of 500-15.6-500 μg/mL after an incubation period of 24 h, hence suggesting that as prepared nanosystem displays small cellular toxicity.

Moreover, it is very important to authenticate the cellular uptake behaviour of synthetic nanocomposites prior their application for in vitro and in vivo MR imaging studies. Herein, HeLa cancerous cells cultured with as-prepared PEGylated Fe_2_O_3_ nanoparticles for 0.5, 1 and 3 h at 37 °C were observed by CLSM photographs Fig. 4b. As we know, DAPI dye emits blue light in the presence of a 488 nm laser, thus it was used to mark the cell nuclei. Besides, PEGylated Fe_2_O_3_ loaded on to the nanocomposite radiates the red emission upon laser irradiation. Hence, the overlay photographs of the above two mentioned channels are shown. And in the pictures at 0.5 h, there is a feeble red fluorescence indicating that only a small fraction of the PEGylated Fe_2_O_3_ has been engulfed by the cells. As the incubation time increases, the red signal intensity increases phenomenally, indicating that the enhanced number of nanorods located in the cells. Thus, these results authenticate our synthesized nanorods can be easily internalized by HeLa cells.

### In vitro photothermal cancer studies

**Fig. 5:**
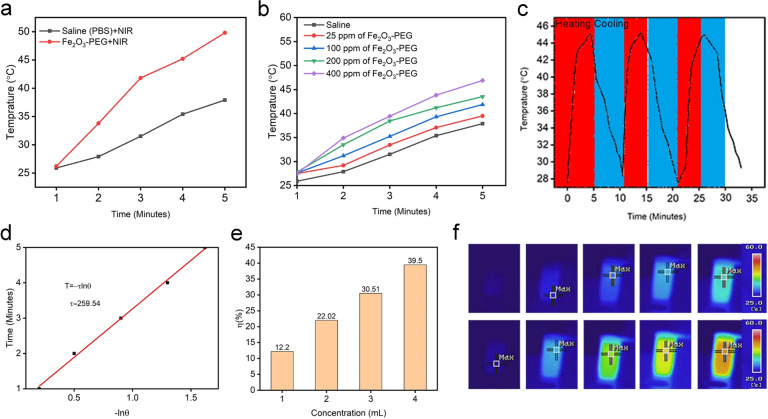
**a** Temperature increase with time for α-Fe_2_O_3_-PEG and PBS (**b**) Increase in the temperature of α-Fe_2_O_3_-PEG at different concentrations plotted against time, (**c**) Photothermal stability laser on and laser off cycles, (**d**) Plot of lnθ vs time, (**e**) Photothermal conversion efficiency of α-Fe_2_O_3_-PEG (**f**) NIR thermal images of α-Fe_2_O_3_-PEG and PBS temperature increase after irradiation for 5 minutes with an 808 nm laser at 0.5Wcm^2^

The in vitro photothermal conversion efficiency of the α-Fe_2_O_3-_PEG was determined by irradiation with an 808 nm laser for 5 min at a power density of 0.5 Wcm^−1^. First the photothermal response of α-Fe_2_O_3-_PEG nanorods was determined in comparison with PBS as a control. We observed that there was a steady rise in temperature of α-Fe_2_O_3-_PEG nanorods with time and it reached a maximum of 49.8 °C after 5 min exposure with 808 nm laser as shown in Fig. 5a, recorded with a NIR thermal imaging camera. The PBS as a control showed a slight increase in temperature for first 3 min and remained steady at 34.6 °C. Encouraged by these results we conducted the photothermal studies of α-Fe_2_O_3-_PEG nanorods in a concentration manner against time. We observed that the temperature increase was directly proportional to the concentration of sample with time as shown in the Fig. 5b. In order to determine the photothermal stability of α-Fe_2_O_3-_PEG nanorods a heating and cooling cycle was performed over a period of 10 min which showed high photothermal stability as shown in Fig. 5c.

To further evaluate the photothermal conversion efficiency value (η) of α-Fe_2_O_3-_PEG, 1 mL of α-Fe_2_O_3-_PEG aqueous solution (400 ppm) was continuously exposed by an 808 nm laser (0.5 W/cm^2^) until a steady-state temperature, at which point the laser was removed, and the sample was allowed to cool naturally and a plot of lnθ vs time as plot as shown in Fig. 5d. On the basis of the quantification method and detailed calculation of the η value was presented in Supporting Information and calculated to be 39.5 % Fig. 5e, which is higher than most of the iron oxide photothermal agents to the best of our knowledge.

To further confirm the photothermal conversion effect of α-Fe_2_O_3_-PEG. The NIR thermal images of the α-Fe_2_O_3-_PEG and PBS were taken with a NIR digital thermal imaging camera. As shown in the Fig. 5f, the increase in the temperature in the PBS is not as high as the color is in the bluish and green region after 5 min of exposure with NIR. While as in case of α-Fe_2_O_3-_PEG the color changes from the blue to red with time depicting an increase in temperature with time. Thus, from above results we can confirm our α-Fe_2_O_3-_PEG can be used as an effective PTT agent for photothermal therapy.

**Fig. 6:**
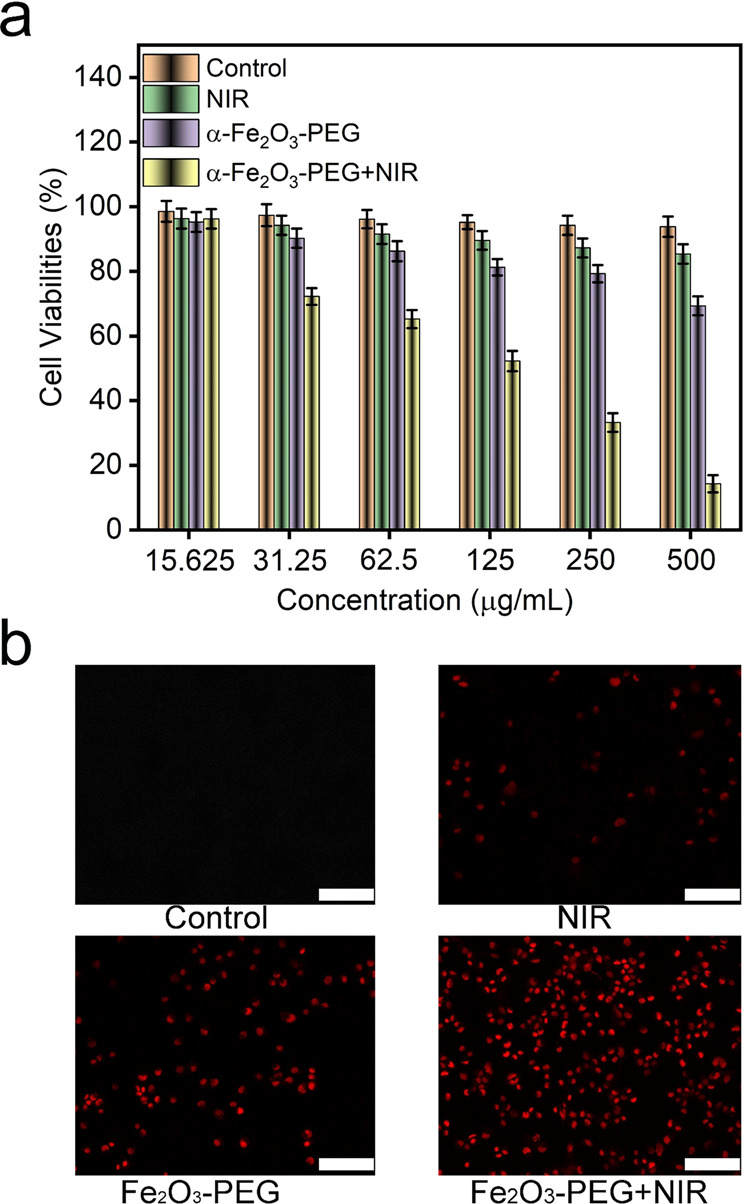
**a** In vitro MTT Cellular assay for cytotoxicity of different treatment groups, Statistical analysis was performed using Student’s two-tailed *t* test (*P<0.05) (**b**) Am/PI double dye stained. Scale bar 50 µm

Encourage by the above results we conducted the in vitro photothermal cancer therapeutic studies of our α-Fe_2_O_3-_PEG nanorods by employing cancerous Hela cells. Hela cells were cultivated in a 6-well plate for 24 h in an incubator at 5% CO_2_. The cytotoxicity’s of different samples were detected by employing the standard methyl thiazolyl tetrazolium (MTT) assay. The HeLa cells were incubated with NIR treatment groups as follows: Control, NIR, α-Fe_2_O_3_, and α-Fe_2_O_3-_PEG+NIR. The concentrations of α-Fe_2_O_3_, and α-Fe_2_O_3-_PEG were set as 15.63, 31.5, 62.5, 125, 250, and 500 µg mL^−1^. Here we observed negligible cell toxicity in the cells treated with NIR. The cell survivability was almost in the rage of 95–73% in the group treated with α-Fe_2_O_3-_PEG devoid of NIR treatment. There was an excellent tumor cell killing observed in the 4th group treated with α-Fe_2_O_3-_PEG+NIR Fig. 6a. This excellent result can be attributed to the photothermal conversion ability of α-Fe_2_O_3-_PEG when irradiated with a NIR laser for 5 min at a power density of 0.5Wcm^2^. From these results we can confirm our nanorods can act as excellent photothermal therapeutic agents for PTT.

The PTT treatment of Fe_2_O_3-_PEG+NIR was also ensured by calcein AM and propidium iodide (PI) double-staining method in Fig. 6b, in which the live cells were stained by green color and the dead cells were red. For groups treated with pure NIR or α-Fe_2_O_3-_PEG minus NIR, there was no obvious damage to the HeLa cells. Then, the α-Fe_2_O_3-_PEG+NIR treated group exhibited a high cytotoxicity with almost 90% dead cells Fig. 6b. The result from the AM-PI method was matched with the cytotoxicity experiment of α-Fe_2_O_3-_PEG+NIR.

### In vitro and in vivo MR imaging property of α-Fe_2_O_3_

**Fig. 7:**
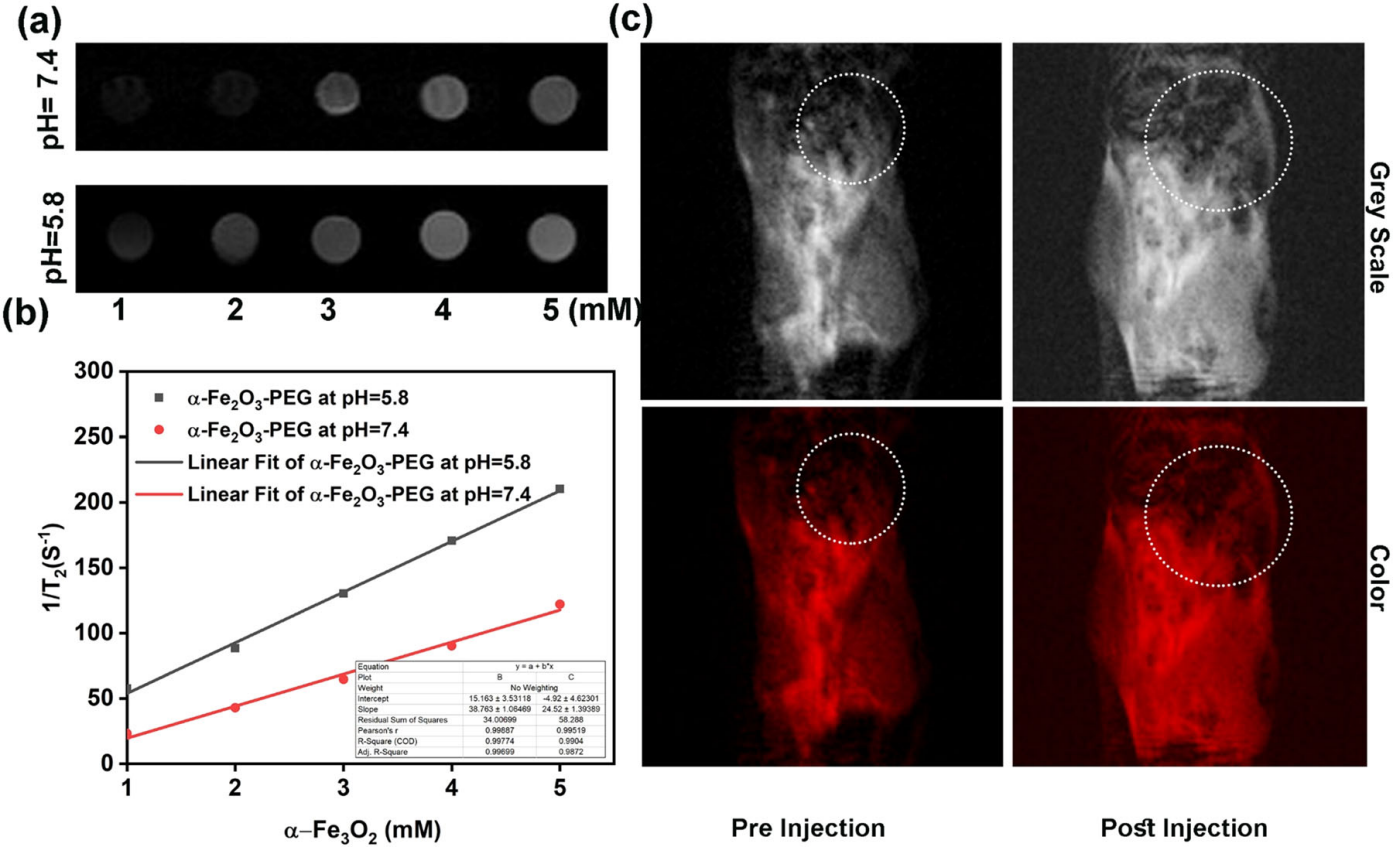
**a** In vitro MR imaging effect of α- Fe_2_O_3_-PEG at pH = 7.4 and 5.8. **b** The MRI concentration varied relaxation rate *r*_*2*_ α- Fe_2_O_3_-PEG at pH = 7.4 and 5.8. **c** In vivo MR imaging effect of α- Fe_2_O_3_-PEG pre and post injection

As per previous studies, the Fe^3+^ ions display a positive enhancement of *T*_*2*_ MR imaging signal. Due to hypoxic tumor environment the Fe_3_O_2_ nanorods undergo Fenton reaction to give rise to Fe^3+^ ion in presence of H_2_O_2_. So, we envisioned the idea of a nanorods for excellent MR imaging outcome. Herein we investigated the *T*_*2*_-weighted MR imaging effect of PEGylated Fe_2_O_3_ dissolved in PSB at pH = 7.4 and 5.8. The in vitro *T*_*2*_-weighted images display a pH-responsive concentration-dependent whitening effect Fig. 7a. We can observe that the concentration dependent whitening effect at pH 5.8 was higher in comparison to that of pH = 7.4 because of the decomposition of PEGylated Fe_2_O_3_ into Fe^3+^ ions at pH = 5.8, which was also illustrated in Fig. 7a. Furthermore, it was found that relaxation *r*_*2*_ (1/*T*_*2*_) signal displays a linear increase with the total concentration of Fe^3+^ varying from 1 to 5 mM at two different pH values Fig. 7b. The relaxation rate (*r*_*2*_ value) at pH = 5.8 was estimated to be 38.763 mM^–1^ s^–1^, which is phenomenally higher than the relaxation rate (*r*_*2*_ value) of 24.52 at pH = 7.4. Hence PEGylated Fe_3_O_2_ can create the MR contrast on a transverse photon relaxation-time-weighted sequence to successfully shorten the *T*_*2*_ relaxation time.

**Fig. 8:**
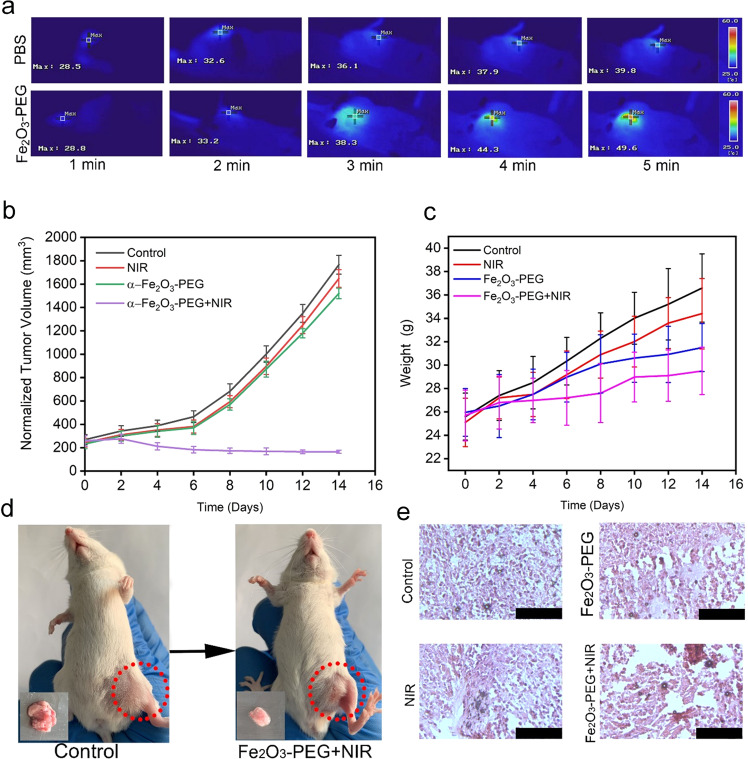
**a** NIR thermal images of mice treated with PBS and α-Fe_2_O_3-_PEG, (**b**) Normalized tumor volume of different treatment groups, (**c**) Body weight of different treatment groups, (**d**) digital photographs of mice, (**e**) H&E stained tissue of tumors from different treatment groups. Scale bar 100 µm. Statistical analysis was performed using Student’s two-tailed *t* test (*P<0.05)

Encouraged by the above results we performed the in vivo *T*_*2*_-weighted MR imaging studies on tumor-bearing mice. As revealed in Fig. 8c, there is a clear *T*_*2*_-MR signal attenuation effect for the mouse with sample injection in comparison to the tumor-bearing mouse without sample injection, signifying the extraordinary potential of Fe_2_O_3_ nanorods as a *T*_*2*_-MR imaging contract agent.

### In vitro and in vivo photothermal cancer therapy

Encouraged by the excellent in vitro photothermal therapeutic results we envisioned the idea of exploring the photothermal therapeutic effect of α-Fe_2_O_3_ nanorods in vivo. Two female balb mice were intravenously injected with saline and α-Fe_2_O_3_-PEG. The tumor sites were irradiated with 808 nm NIR laser at a power density of 0.5 Wcm^2^ for a time period of 5 minutes and the temperatures were recorded with a digital NIR thermal imaging camera as show in Fig. 8a.

As we observe here that the temperature reached a maximum of 39.8 °C in the mice injected with PBS over a time of 5 min after exposure with an 808 nm laser at power density of 0.5 Wcm^2^. While as in case of the mice injected with α-Fe_2_O_3_-PEG the temperature raised up sharply over a time periods of 5 min to 49.6 °C thus confirming excellent in vivo photothermal conversion ability of α-Fe_2_O_3_-PEG nanorods under NIR irradiation.

**Fig. 9:**
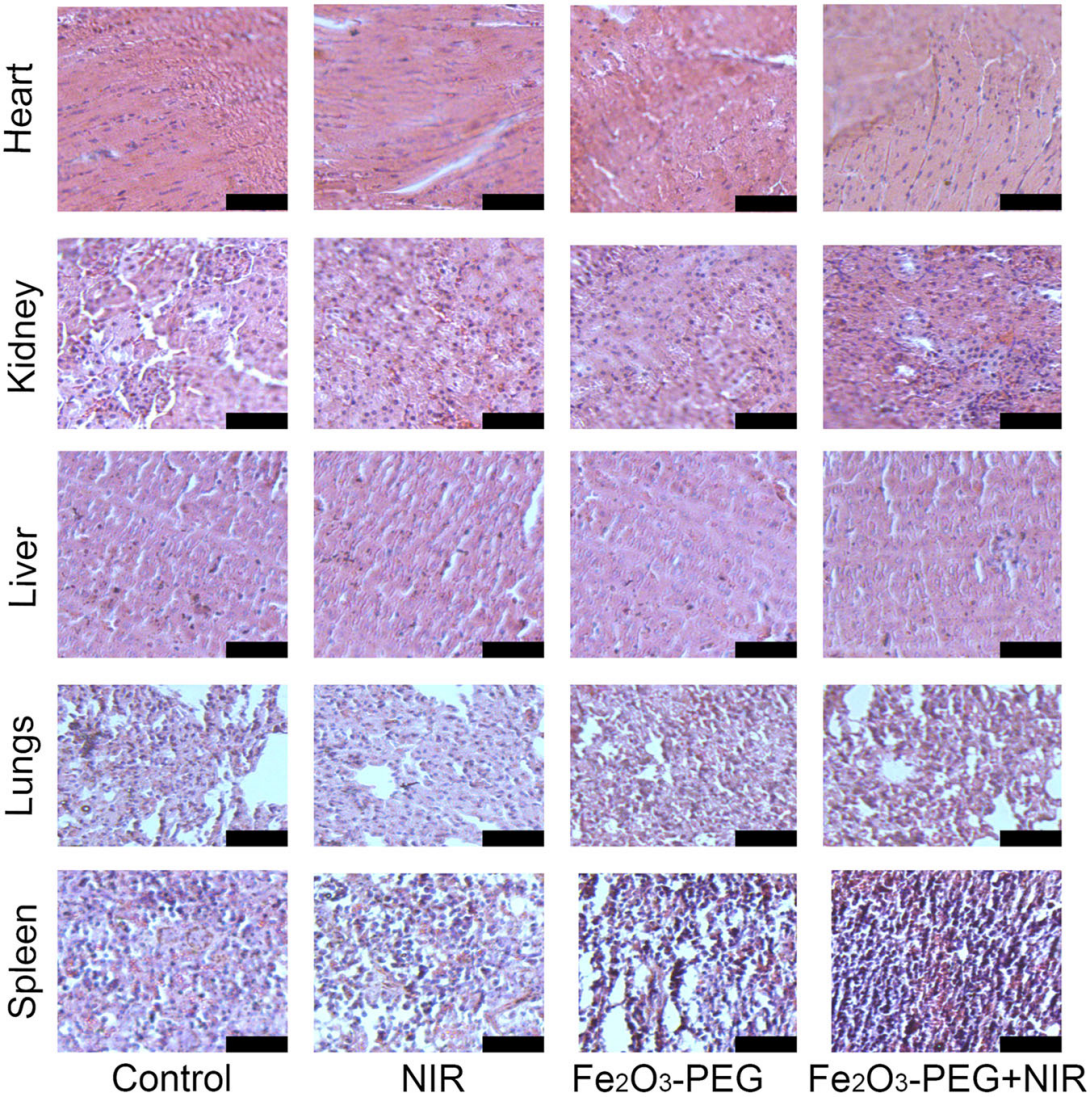
H&E staining of vital organs. Scale bar 75 µm

Encouraged by the above photothermal conversion ability of α-Fe_2_O_3_-PEG we conducted a thorough in vivo study of the PTT property of α-Fe_2_O_3_-PEG. Female balb mice were divided into four groups (*n* = 5). The group 1st was left untreated as control, while as the group 2nd was treated with only NIR. The 3rd group was treated with α-Fe_2_O_3_-PEG without NIR irradiation. Finally, the 4th group was treated with α-Fe_2_O_3_-PEG+NIR with 808 nm laser at power density of 0.5 Wcm^2^. Post tumor bearing except for the control group the mice in 2nd were irradiated with NIR light for 5 min for two weeks, and mice in 3rd group were injected with α-Fe_2_O_3_-PEG and left for rest of the two weeks for observation. The 4th group mice were injected with α-Fe_2_O_3_-PEG and treated with NIR laser for 5 min daily for two weeks. The representative tumor volume and body weight for different treatment groups can be seen from the Fig. 8b, c. From the Fig. 8d we can see there is a considerable reduction in tumor from the control group to the final treatment group. The extracted tumor weight was recorded and it was found that tumor size decreased from 2.1 grams in the control group to 0.4 grams final treatment group Fig. S6a. The survival rates showed that the mice treated with α-Fe_2_O_3_-PEG and treated with NIR laser for 5 min daily for two weeks survived for over 35 days Fig. S6b. H&E staining of the tumor tissue from different treatment groups are shown in the Fig. 8e. We can see there is no damage to the tumor tissue in the control group, while as a minor aberration is seen in 2nd and 3rd group. There is a significant tumor tissue damage in the final group. Thus, confirming the excellent in vivo PTT therapeutic potential of α-Fe_2_O_3_-PEG nanorods. In Fig. 9, the H&E-stained images of the main organs including spleen, lung, heart, liver, and kidney extracted from groups I–V presented that there were no obvious injury and necrosis.

## Conclusion

α-Fe_2_O_3_ nanorods were successfully fabricated by calcination of hydrothermally synthesized goethite (FeOOH) nanorods. Structural and optical properties have been discussed in detail. Un-annealed sample (goethite) showed compressive strain and annealed sample showed tensile strain. This change is due to calcination at 500 °C. The annealing leads goethite to convert into hematite phase. The hematite nanorods exhibited high visible bandgap which makes them an important candidate in various optical and biomedical applications such as PTT and MRI. A significantly low cytotoxicity and high cellular uptake of our as prepared PEGylated nanorods confirmed their potential use in the biomedical field. A high photothermal conversion efficiency was achieved besides a high MRI *T*_*2*_ relaxation rate was observed both in vitro and in vivo further enhancing the prospect of using α-Fe_2_O_3_ biomedical imaging research. Our study explores the potential of using α-Fe_2_O_3_ for various cancer theranostic studies.

## Supplementary Information

Electronic Supplementary Information

Revised-ESI
