# Supplementary material for: In vitro and in vivo MRI imaging and photothermal therapeutic properties of Hematite (α-Fe2O3) Nanorods
Source: J Mater Sci Mater Med. 2022 Jan 12;33(1):10. doi: 10.1007/s10856-021-06636-1 (PMC8755697; doi:10.1007/s10856-021-06636-1)
Supplement: Supplementary file 1 — Electronic Supplementary Information [file 10856_2021_6636_MOESM1_ESM.docx]

**Supporting Information**

*In vitro* and *In vivo* MRI imaging and photothermal therapeutic properties of Hematite(α-Fe_2_O_3_) Nanorods

*Aanisa Gulzar,^a1^ Nowsheena Ayoub,^a1^ Jaffar Farooq Mir, ^a^ M A Shah, ^a*^ Arif Gulzar, ^b*^*

^a^*Department of Physics, National Institute of technology, Srinagar, J&K,190006, India*

*^b^MedX institute, College of Biomedical Engineering, Shanghai Jiaotong University. Shanghai,200030, PRC.=*

E-mail: [arifgulzar@sjtu.edu.cn](mailto:arifgulzar@sjtu.edu.cn), Shah@nitsri.ac.in


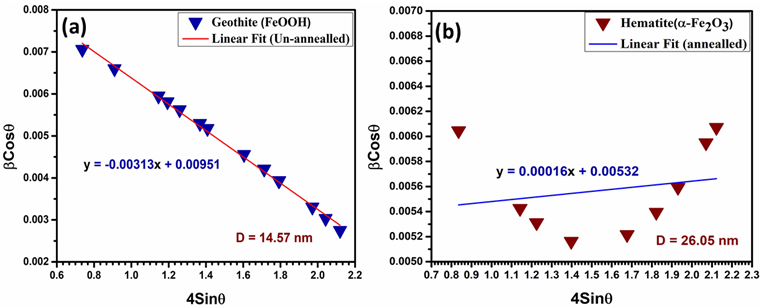


Fig.S1. (Williamson Hall Plot: Goethite (Unannealed sample) 500^o^C(a). Hematite (annealed) sample (b).


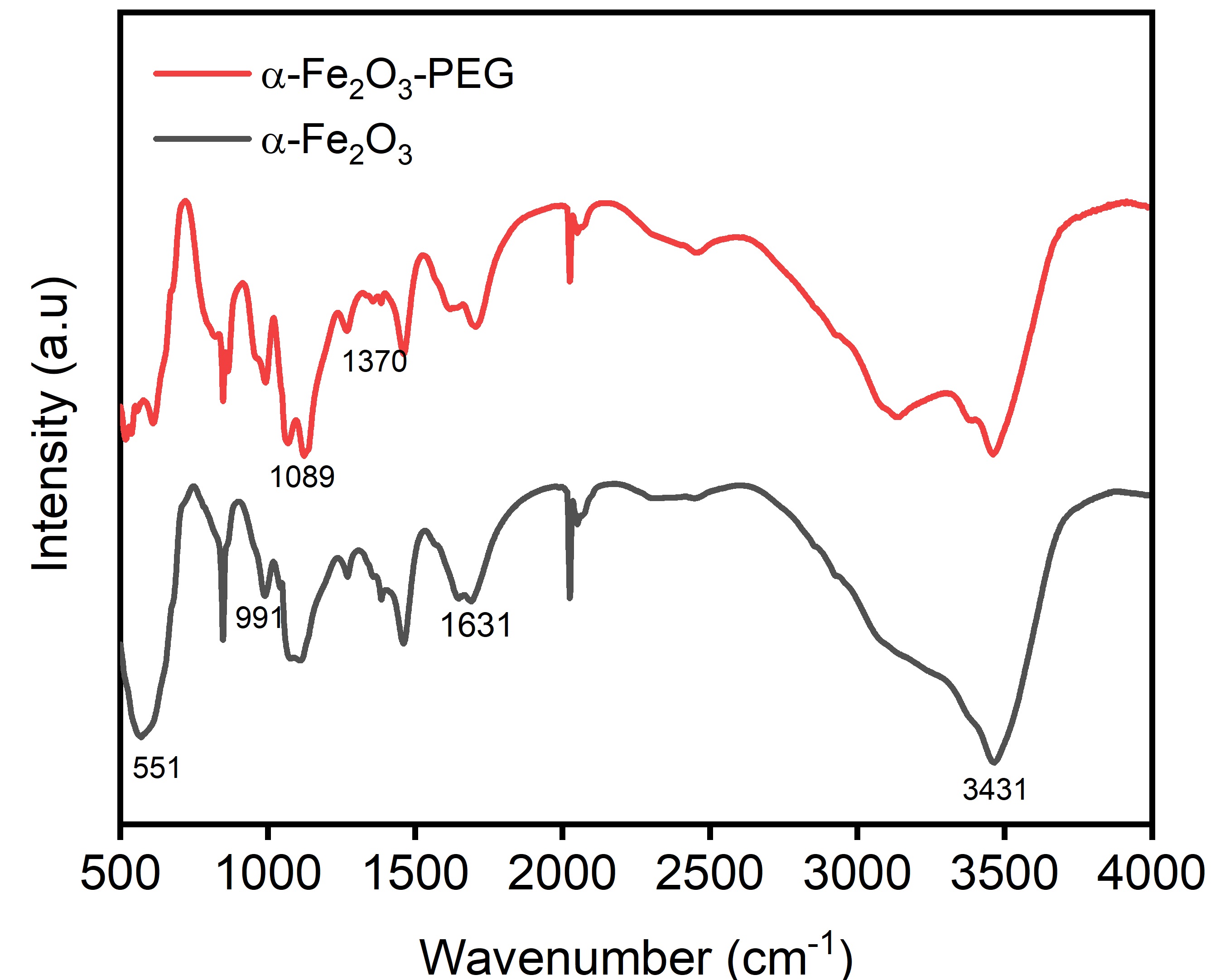


Fig.S2. FT-IR of α- Fe_2_O_3_ and α- Fe_2_O_3_-PEG.


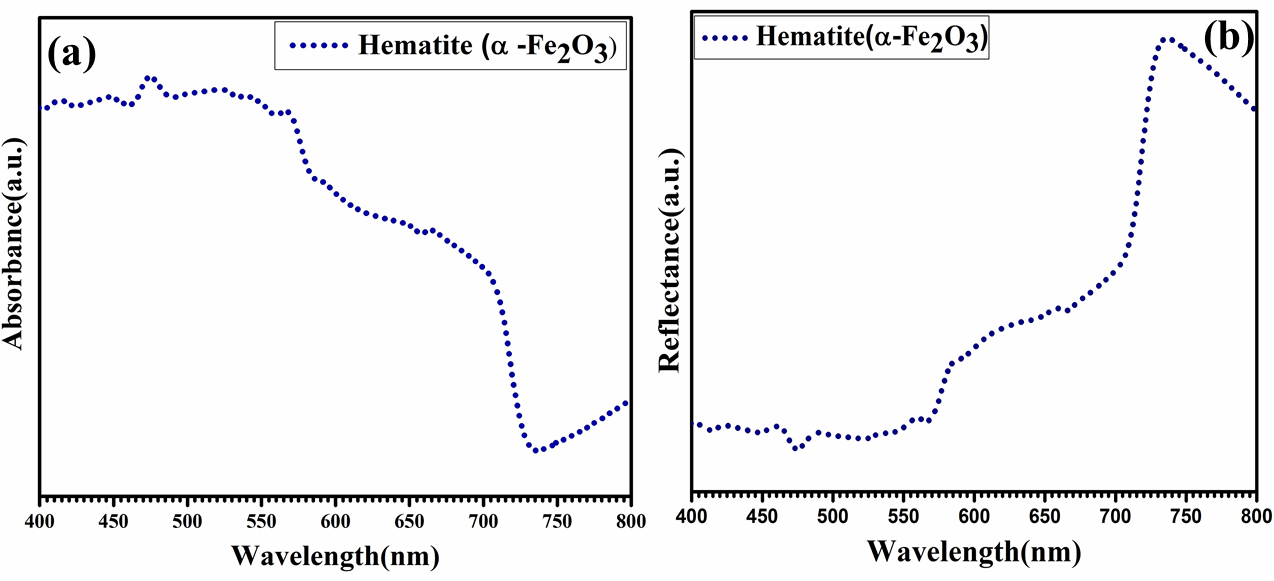


Fig.S3. UV- vis spectra of the hematite nanorods**: (a)** Absorption spectra **(b)**Reflectance spectra.


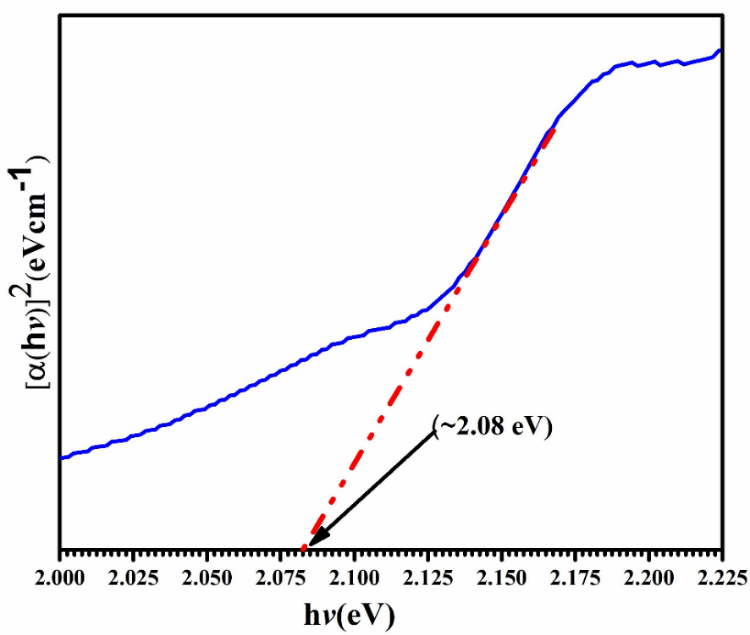


Fig.S4. Tauc plot showing band gap energy of hematite nanorods.


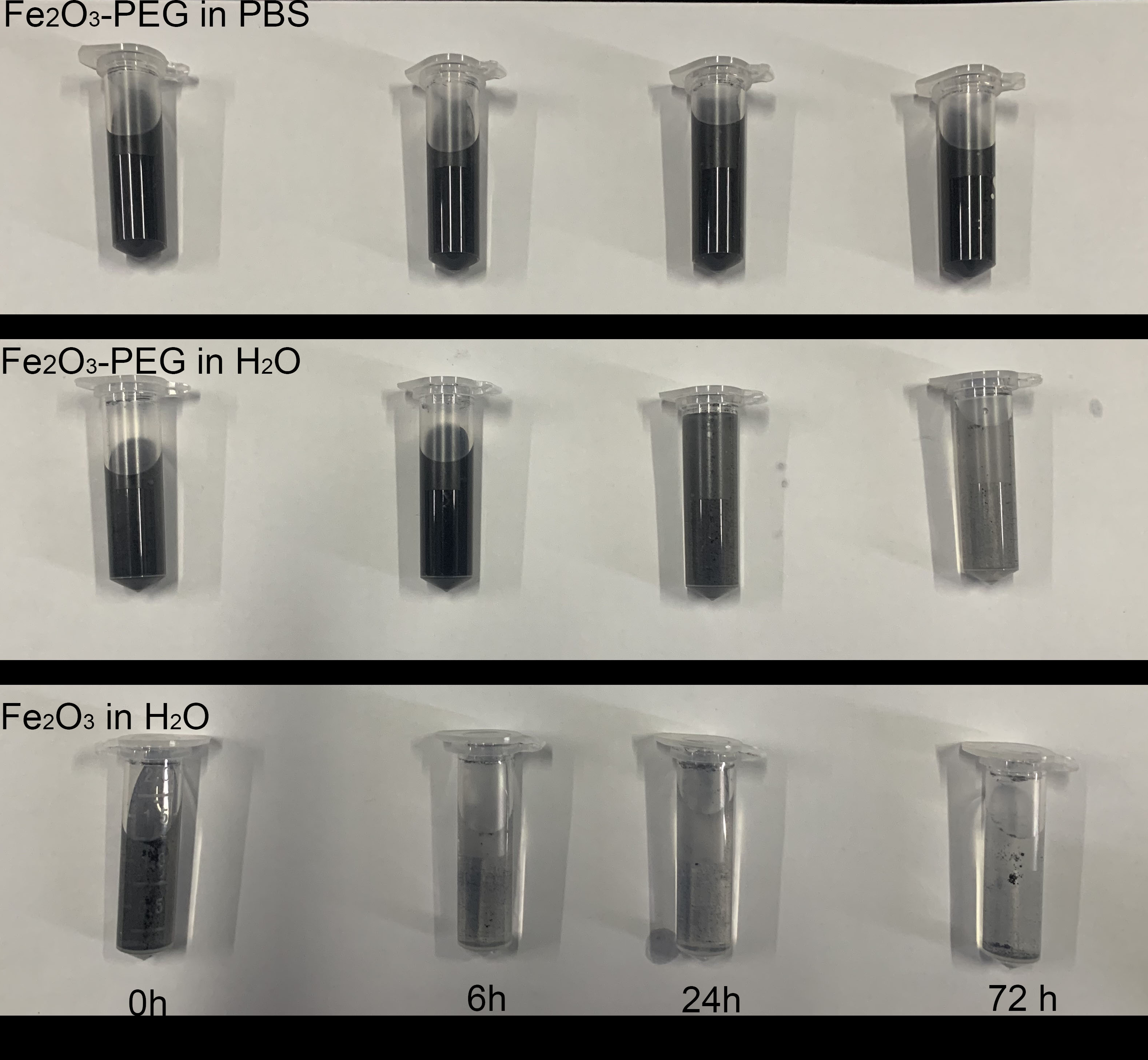


Fig.S5. Solubility of α-Fe_2_O_3_ and α-Fe_2_O_3_-PEG in water and PBS over a time period of 72 hours

Calculation of Photothermal conversion efficiency

Photothermal conversion efficiency of the α-Fe_2_O_3-_PEG was calculated by recording the change in the temperature of the NP aqueous dispersion as a function of time under continuous irradiation of a 808 nm laser (0.5 W/cm^2^) for 5 mins until the solution reached a steady-state temperature. The photothermal conversion efficiency (*ƞ*) was calculated according to Equation S1:

 Equation S1.

Where, h represents the heat transfer coefficient, A is the surface area of the container, T_max_ represents the maximum steady-state temperature (60 °C), T_surr_ is the ambient temperature of the environment (25 °C), Q_dis_ represents the heat dissipation from the light absorbed by the solvent and the quartz sample cell, I is the incident laser power (0.5 W/cm^2^), and A660 is the absorbance of the sample at 740 nm (1.776). The value of hA is derived from Equation S2:

Equation S2.

Where τ is the time constant for heat transfer of the system which was determined to be τ =390.96 from Fig 6d. m*_D_* and c*_D_* are respectively the mass (1.0 g) and heat capacity (4.2 J/g) of the deionized water used to disperse the α-Fe_2_O_3-_PEG. So, the hA was determined to be 0.0201 W. Q_dis_ represents the heat dissipation from the light absorbed by the water and the quartz sample cell, so Q_dis_ was calculated according to Equation S3:

 Equation S3.

Where T_max_(water)is 26.8 °C, τ (water) is 259.25, so Q_dis_ was calculated to be 0.0211 W.

According to the obtained data and Equation (1), the photothermal conversion efficiency of the α-Fe_2_O_3-_PEG was determined to be 39.5%.
