# Supplementary material for: In vitro and in vivo MRI imaging and photothermal therapeutic properties of Hematite (α-Fe2O3) Nanorods
Source: J Mater Sci Mater Med. 2022 Jan 12;33(1):10. doi: 10.1007/s10856-021-06636-1 (PMC8755697; doi:10.1007/s10856-021-06636-1)
Supplement: Supplementary file 2 — Revised-ESI [file 10856_2021_6636_MOESM2_ESM.docx]

**Supporting Information**

*In vitro* and *In vivo* MRI imaging and photothermal therapeutic properties of Hematite(α-Fe_2_O_3_) Nanorods

Aanisa Gulzar,^a1^ Nowsheena Ayoub,^a1^ Jaffar Farooq Mir, ^a^ Amer M Alamazi,^b^ M A Shah, ^a*^ Arif Gulzar, ^c,d^*

^a^*Department of Physics, National Institute of technology, Srinagar, J&K,190006, India*

^b^ *Pharmaceutical Chemistry Department, College of Pharmacy , King Saud University, Riyadh 11451, Saudi Arabia.*

*^c^MedX institute, College of Biomedical Engineering, Shanghai Jiaotong University. Shanghai,200030, PRC.*

^d^ *Hevesy Laboratory, DTU Health Tech, 4000, Roskilde, Denmark*

E-mail: [arifgulzar@sjtu.edu.cn](mailto:arifgulzar@sjtu.edu.cn), Shah@nitsri.ac.in


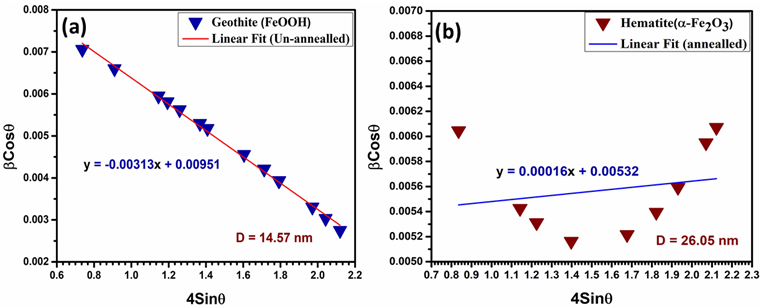


Fig.S1. (Williamson Hall Plot: Goethite (Unannealed sample) 500^o^C(a). Hematite (annealed) sample (b).


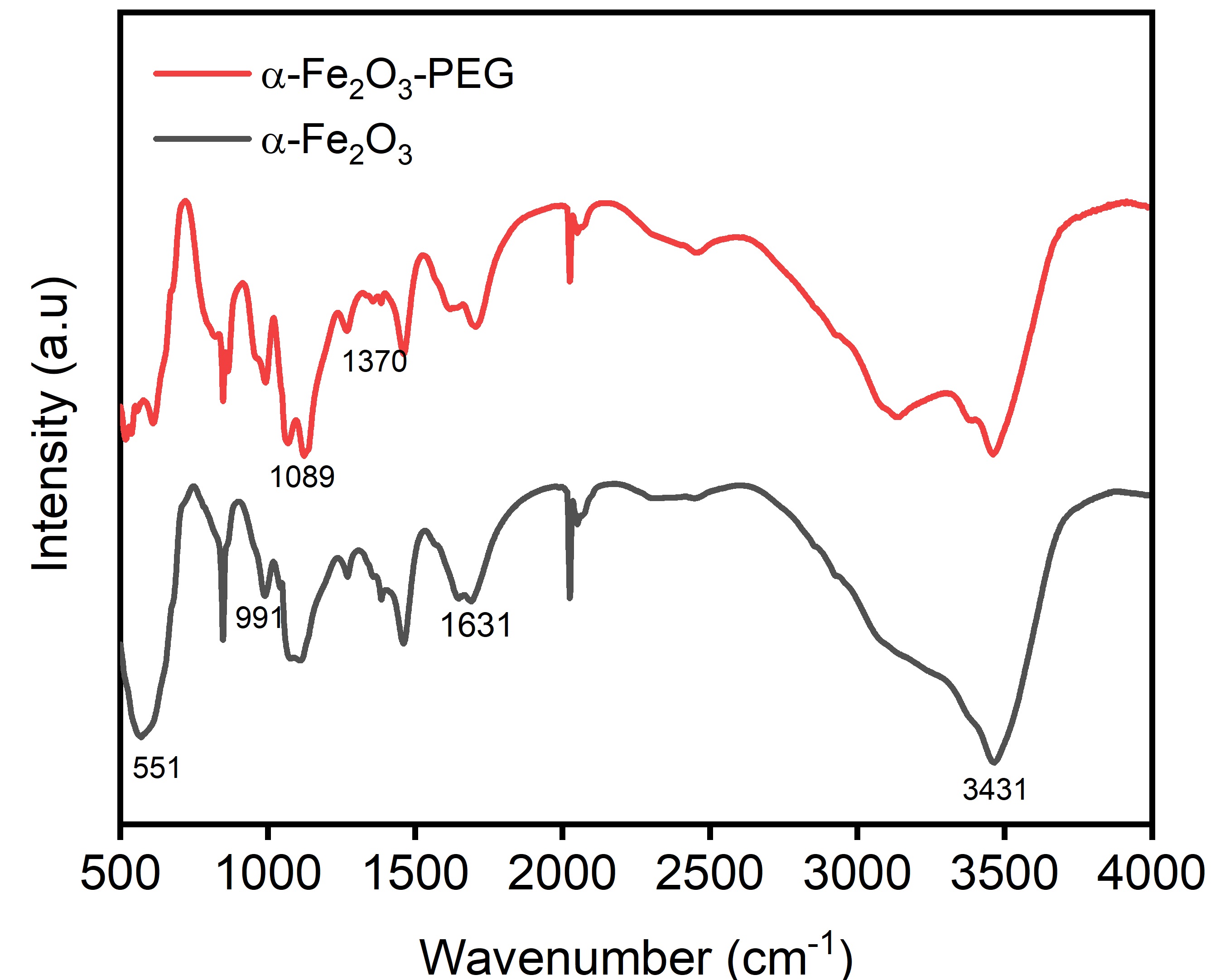


Fig.S2. FT-IR of α- Fe_2_O_3_ and α- Fe_2_O_3_-PEG.


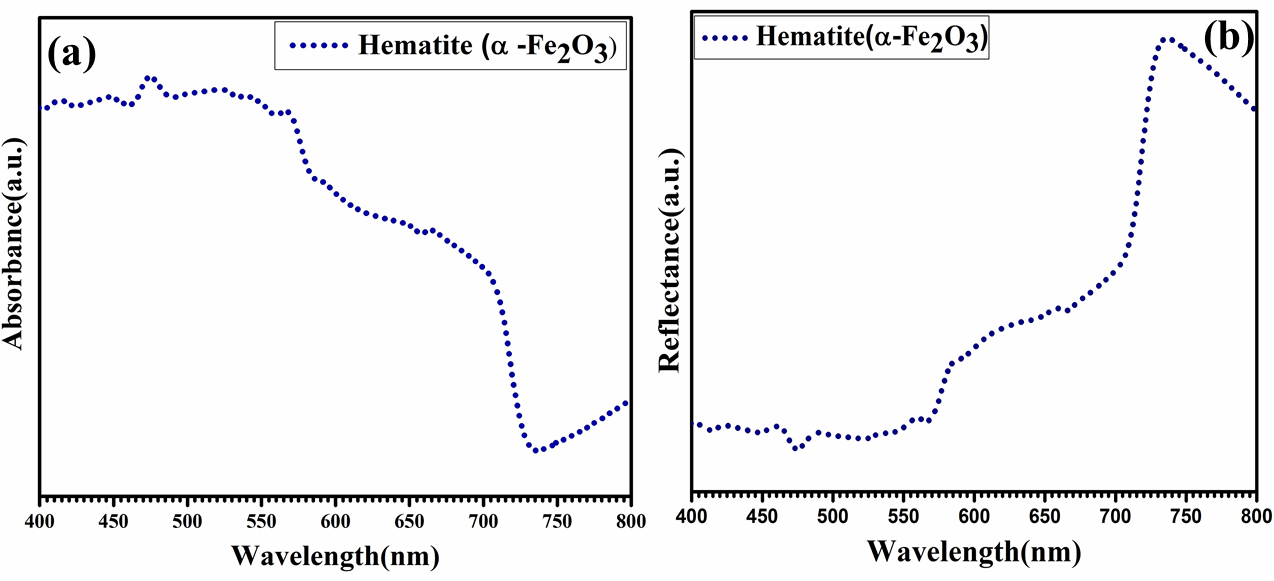


Fig.S3. UV- vis spectra of the hematite nanorods**: (a)** Absorption spectra **(b)** Reflectance spectra.


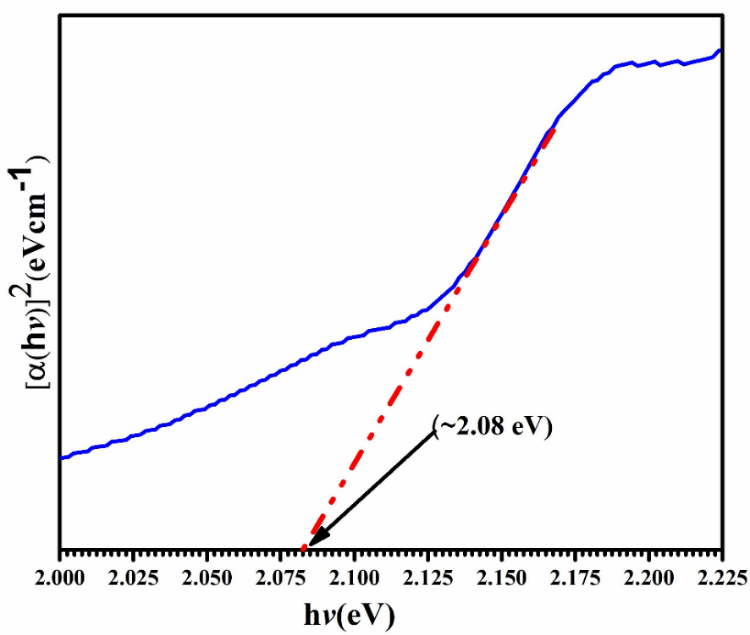


Fig.S4. Tauc plot showing band gap energy of hematite nanorods.


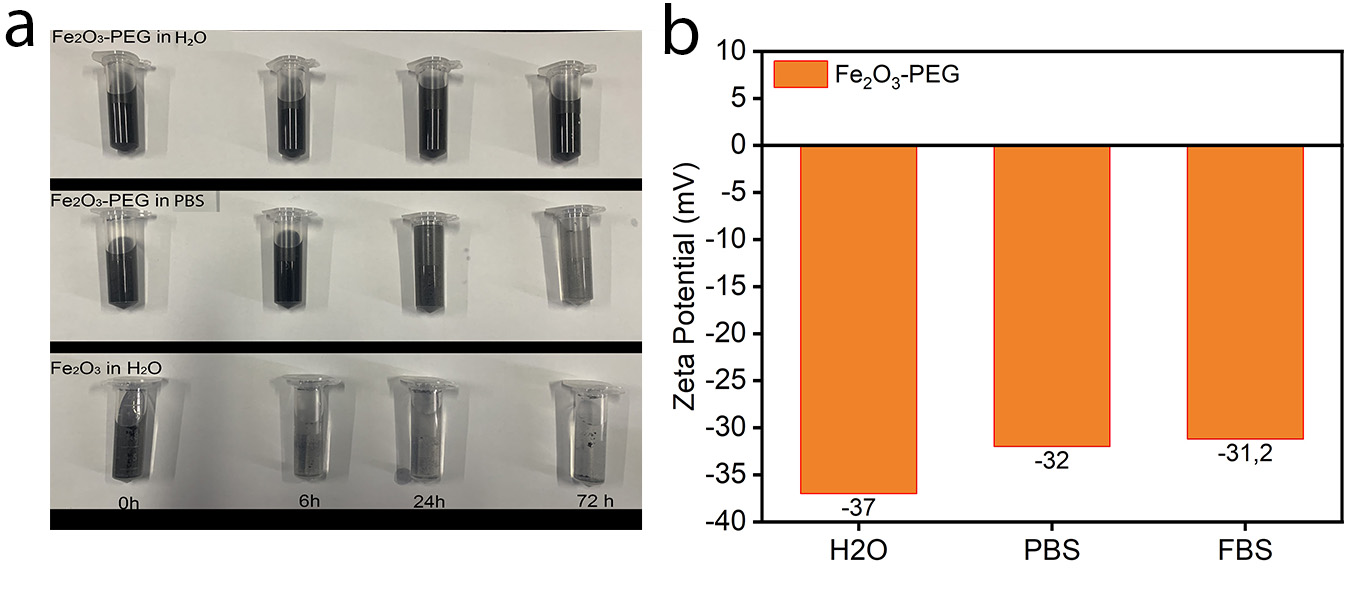


Fig.S5. (a)Solubility of α-Fe_2_O_3_ and α-Fe_2_O_3_-PEG in water and PBS over a time period of 72 hours (b) Zeta potential of α-Fe_2_O_3_-PEG in water, PBS and FBS.


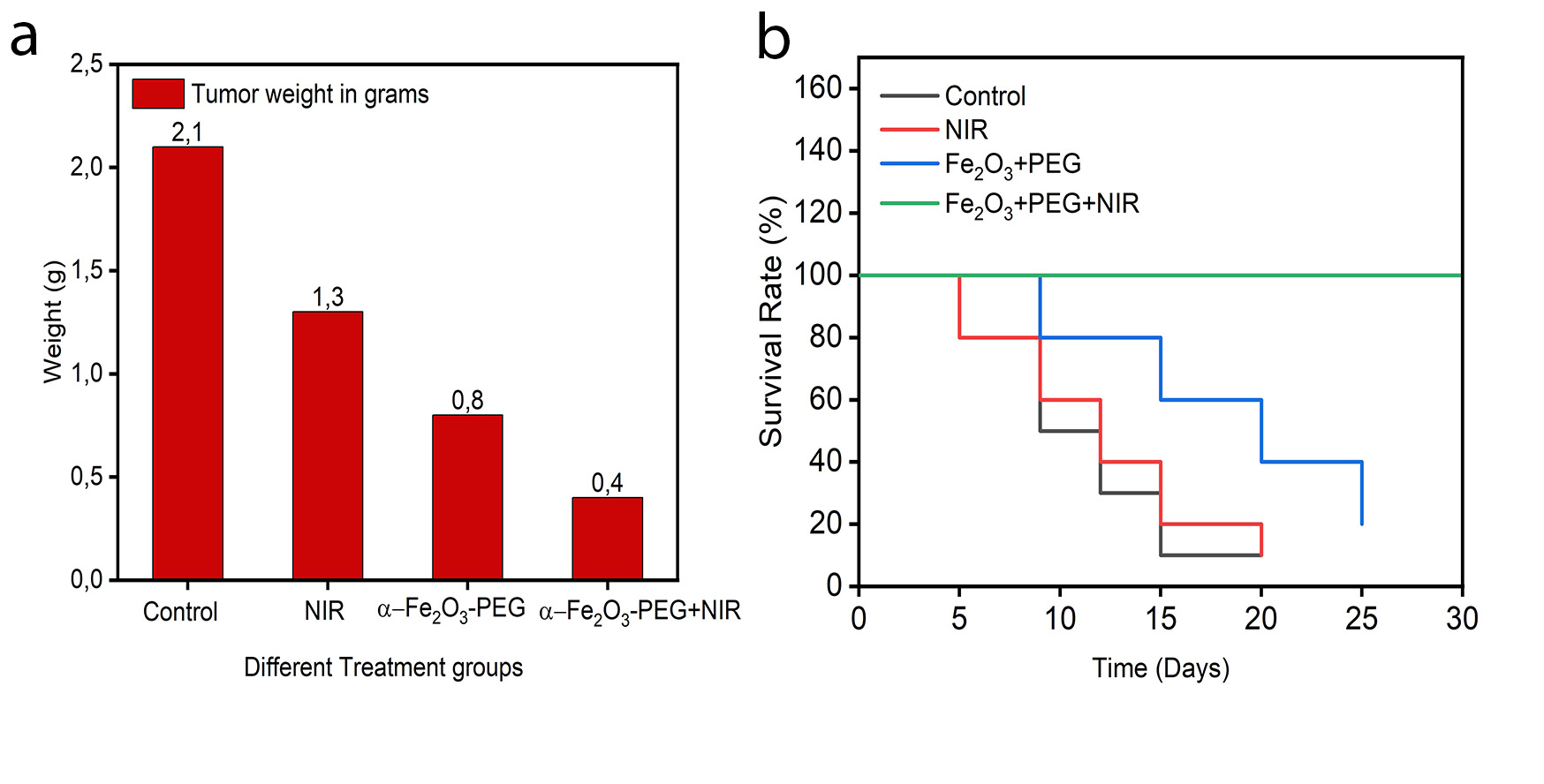


Fig.6S (a) Tumor weight after extraction of different treatment groups, (b) Survival rate of various treatment groups.

Calculation of Photothermal conversion efficiency

Photothermal conversion efficiency of the α-Fe_2_O_3-_PEG was calculated by recording the change in the temperature of the NP aqueous dispersion as a function of time under continuous irradiation of a 808 nm laser (0.5 W/cm^2^) for 5 mins until the solution reached a steady-state temperature. The photothermal conversion efficiency (*ƞ*) was calculated according to Equation S1:

 Equation S1.

Where, h represents the heat transfer coefficient, A is the surface area of the container, T_max_ represents the maximum steady-state temperature (60 °C), T_surr_ is the ambient temperature of the environment (25 °C), Q_dis_ represents the heat dissipation from the light absorbed by the solvent and the quartz sample cell, I is the incident laser power (0.5 W/cm^2^), and A660 is the absorbance of the sample at 740 nm (1.776). The value of hA is derived from Equation S2:

Equation S2.

Where τ is the time constant for heat transfer of the system which was determined to be τ =390.96 from Fig 6d. m*_D_* and c*_D_* are respectively the mass (1.0 g) and heat capacity (4.2 J/g) of the deionized water used to disperse the α-Fe_2_O_3-_PEG. So, the hA was determined to be 0.0201 W. Q_dis_ represents the heat dissipation from the light absorbed by the water and the quartz sample cell, so Q_dis_ was calculated according to Equation S3:

 Equation S3.

Where T_max_(water)is 26.8 °C, τ (water) is 259.25, so Q_dis_ was calculated to be 0.0211 W.

According to the obtained data and Equation (1), the photothermal conversion efficiency of the α-Fe_2_O_3-_PEG was determined to be 39.5%.
